# Remote Monitoring for the Management of Spasticity: Challenges, Opportunities and Proposed Technological Solution

**DOI:** 10.1109/OJEMB.2024.3523442

**Published:** 2024-12-30

**Authors:** Kavit R. Amin, Samuel R. Smith, Amit N. Pujari, Syed Ali Raza Zaidi, Robert Horne, Atif Shahzad, Christopher Walshaw, Christy Holland, Stephen Halpin, Rory J. O'Connor

**Affiliations:** University of Manchester 5292 M13 9PL Manchester U.K.; Manchester University NHS Foundation Trust 5293 M13 9WL Manchester U.K.; Neu(RAL)2: NeuRAL Systems & Rehabilitation and Assistive Technologies Laboratory, School of PhysicsEngineering and Computer ScienceUniversity of Hertfordshire 3769 AL10 9EU Hatfield U.K.; School of EngineeringUniversity of Aberdeen 1019 AB24 3FX Aberdeen U.K.; University of Leeds 4468 LS2 9JT Leeds U.K.; University of Kent 2240 CT2 7NZ Canterbury U.K.; University of Birmingham 1724 B15 2TT Birmingham U.K.; Leeds Teaching Hospitals NHS Trust 4472 LS9 7TF Leeds U.K.; Leeds Teaching Hospitals NHS Trust 4472 LS9 7TF Leeds U.K.; Academic Department of Rehabilitation MedicineUniversity of Leeds 4468 LS2 9JT Leeds U.K.; Academic Department of Rehabilitation MedicineUniversity of Leeds 4468 LS2 9JT Leeds U.K.; NIHR Devices for DignitySheffield Teaching Hospitals NHS Trust 7318 S10 2JF Sheffield U.K.

**Keywords:** Electromyography, rehabilitation, spasticity management, spasticity monitoring, wearable device

## Abstract

Spasticity is disabling feature of long-term neurological conditions that has substantial impact on people’ quality of life. Assessing spasticity and determining the efficacy of current treatments is limited by the measurement tools available in clinical practice. We convened an expert panel of clinicians and engineers to identify a solution to this urgent clinical need. We established that a reliable ambulatory spasticity monitoring system that collates clinically meaningful data remotely would be useful in the management of this complex condition. This paper provides an overview of current practices in managing and monitoring spasticity. Then, the paper describes how a remote monitoring system can help in managing spasticity and identifies challenges in development of such a system. Finally the paper proposes a monitoring system solution that exploits recent advancements in low-energy wearable systems comprising of printable electronics, a personal area network (PAN) to low power wide area networks (LPWAN) alongside back-end cloud infrastructure. The proposed technology will make an important contribution to patient care by allowing, for the first time, longitudinal monitoring of spasticity between clinical follow-up, and thus has life altering and cost-saving implications. This work in spasticity monitoring and management serves as an exemplar for other areas of rehabilitation.

## I. Introduction and Healthcare Need

Spasticity is a feature of upper motor neuron condition that arises from lesions in the central nervous system. It is characterized by increased tonic stretch reflexes resulting in velocity and length-dependent hypertonia due to abnormal spinal processing of proprioceptive input. This clinical scenario is commonly seen in people living with cerebral palsy (CP; 90%), acquired brain injury (ABI; 17–38%), spinal cord injury (SCI; 40–78%), multiple sclerosis (MS; 50%) and some neurodegenerative conditions [1], [2].

Impairments can vary from mild tightness to joint immobility. In the upper limb, the shoulder adductors, elbow, wrist and finger flexors are commonly involved. In the lower limb, variable impacts on hip adductors, knee, plantar flexors, ankle invertors and toe extensors can alter posture and gait. Function is limited by the observed variability in clonus, spastic co-contraction, spasms (sudden involuntary movement, which can be painful), dystonia [3] and associated reactions [4]. Spasticity can also mask volitional movement in antagonistic muscles. The sequelae can be disabling, affecting activities of daily living such as eating, personal hygiene and dressing, resulting in increasing care needs. Impact on posture causes an increased susceptibility to pressure ulcers [5]. Untreated, in its severest forms, spasticity can result in pain, contractures, and ultimately joint subluxation [6].

The psychological impact of spasticity is not limited to people living with spasticity, with wider impacts on carers and families [7], with substantial costs to health and social care economies. Spasticity can provide clinical benefits. Increased tone can facilitate sitting, standing, walking and transfers. For this reason, the management of spasticity should be goal-focused to personalize treatments through minimizing the impact of the condition whilst not taking away any potential benefit.

### A. Current Practice in Managing Spasticity Al

The evidence base for the management of spasticity is limited, with the U.K. National Institute for Health and Care Excellence (NICE) guidelines only available for children, with limited guidance for the treatment of adults [8]. Healthcare professionals have highlighted priority areas for improving patient care, in particular, the lack of standardized treatment protocols and validated measurement tools [9].

In principle, management aims to normalize tone where feasible. Patients should be under regular review by clinical teams with a specialist interest in spasticity [10]. However, access to specialist clinics remains varied across the U.K. and has been further impacted by the COVID-19 pandemic. It can be inferred that earlier identification and management of spasticity could limit secondary long-term complications.

The cornerstone of spasticity management is a multidisciplinary approach, with a range of therapy, pharmacological and surgical options. Therapy broadly includes splinting, stretching, serial casting, direct tendon pressure and functional electrical stimulation (FES) [1]. Pharmacotherapy, in the form of oral antispasticity agents or intramuscular botulinum toxin can be offered. Other approaches include chemical neurolysis with alcohol or ITB, but these carry the risk of axonal injury and dysesthesia, and insertion of an implanted pump to deliver the medication baclofen into the intrathecal space. Surgical treatment options include tendon lengthening, transfers, peripheral neurotomy, rhizotomy and neuroablative procedures.

Although no formal, overall guidelines exist for neurological conditions in general, in the U.K., NICE recommends physiotherapy for MS associated spasticity, and pharmacotherapy for those in pain, discomfort, or facing a loss of dexterity [11]. For post-stroke spasticity, NICE recommends the use of botulinum toxin, pharmacotherapy, physical therapy and splinting [12]. Interestingly, the functional benefit of pharmacotherapy has been questioned in clinical trials, even though there is a reduction in impairment-level tone and pain [13]. A systematic review of 101 studies identified limited evidence for the efficacy of baclofen, tizanidine and dantrolene, and evidence for combination therapy is scarce [14]. The side effect profile of these medications is substantial. Baclofen can also be delivered via an implanted pump infusing into the intrathecal space. Intrathecal phenol is destructive and can damage motor and sensory nerves [15].

Across healthcare there is an increasing onus to steer service provision towards community care [1]. Given the paucity of evidence to support treatment guidelines, a pragmatic, personalized, multidisciplinary approach is a feasible strategy in managing the complexities of this patient group. Research priorities would benefit from modeling progression of spasticity, the efficacy of current interventions and targeting preventative measures that reduce symptoms and long-term complications.

### B. Monitoring Spasticity

There is no consensus on the validity of measures used to stratify and monitor the progression of spasticity. A systematic review evaluating outcomes after acute SCI identified a paucity of research in the early detection of spasticity [16]. In part, this may be due to the myriad of presentations and the accuracy in their validation. Clinical scales include the Ashworth Scale (AS; developed in 1964; republished as the Modified AS (MAS) in 1980), Tardieu, and composite spasticity scales [17]. The MAS is a muscle tone assessment scale used to assess the resistance experienced by an observer during passive range of motion, but has poor validity, reliability, and responsiveness [18]. The Tardieu system assesses the angle of muscle reaction [19]. Patient-reported outcome measures (PROM) such as the arm activity (ArmA) [20] and visual analogue scales (VAS) [21] have been reported.

In the U.K., clinicians typically serially follow-up patients on a 4–6 monthly basis, providing longitudinal snapshots of the clinical condition, but making it challenging to identify functional gains post intervention. The merits of multidisciplinary follow up 4–6 weeks post intervention is recognized in national guidelines [8] but is not widely practiced as it is resource intensive and requires additional health care professional contact. The logistics from the perspective of patients, carers and families in attending additional clinic appointments also warrants consideration.

While attempts have been made to identify reliable measures for spasticity, they are limited. Determining the effect of treatment modalities via biomechanical assessments measuring joint motion and resistance, isokinetic dynamometer and neurophysiological assessments have been suggested [22]. Indeed, the quantification of spastic tone, and its correlation with voluntary muscle movement is an attractive proposition, yet its full utility is unknown [23] and combines surface electromyography (sEMG) with active, passive and involuntary movement [24]. It would be helpful to clinically differentiate between tone of neural and non-neural origin [25] as they require differing treatment strategies. Dynamic electromyography can provide information about muscle group spasticity. However, concerns have been raised by a panel of experts regarding both surface marker systems, which can be inaccurate and require expertise for interpretation, and needle testing, which can be painful and may itself raise limb tone [19].

Newer developments focus on, but are not limited to: clinical scales (numeric rating scale [26], spasticity scale [27]); medical imaging (near-infrared spectroscopy [28], MRI [29], ultrasonography [30] and thermography [31]); gait analysis; neural measurements; and telerehabilitation [32] (driven by the COVID-19 pandemic, examples include motion analysis, quality of life and self-reported spasticity assessments, which may play a role for individuals less able to attend a clinic). It is important to note that most of the above measures provide snapshot assessments, as do clinical measures based on assessments performed by clinicians, such as the MAS. However, spasticity varies over time and is affected by factors such as temperature, posture, circadian rhythm, timing of medication, menstrual cycle and activity [33]. Hence longitudinal monitoring of spasticity is critical.

It is likely that a combined multi-modal approach will be the most effective way to longitudinally assess spasticity as there is unlikely to be a single measure to suit all presentations. The approach to spasticity assessment and management is oriented around the problems an individual is facing, and lie in differing domains (e.g., pain, spasms, or active or passive functional limitation). Therefore, individual assessment and a suite of possible measures will be necessary.

The main strategies for spasticity evaluation include devices such as portable-sensor devices (inertial sensors, sEMG) [34], robot-assisted equipment (may use goniometers, pressure sensors and EMG for use in exoskeletons) [35] and myotonometry whereby a probe sends brief pulses to spastic muscles [36]. They remain limited at the current time given their limited validation, and the expertise and specialist equipment is not readily available for use. Spasticity is more evident with active rather than passive movements and future research should focus on evaluating these in addition to patient related outcome measures (PROMs) to further aid assessment.

## II. Methods

### A. Interdisciplinary Expert Meeting on Spasticity Management

A roundtable discussion was held in Leeds (U.K.) on 23/01/23 with expert clinicians and engineers to: 1) reach a consensus for the long-term monitoring and management of spasticity on clinical grounds and to: 2) consider any prerequisites when developing a technological solution designed for monitoring spasticity. The expert participants included 9 clinicians (6 rehabilitation physicians routinely involved in spasticity management, 1 plastic surgeon, 1 specialist occupational therapist, 1 specialist physiotherapist) and 4 engineers (2 electronics, 1 communication and 1 biomedical engineer). The first half of the discussion centered on the need for long-term monitoring and the current management of spasticity. Synthesis of this part of the discussion has been presented in the ‘Introduction and Healthcare Need’ section above. The second half of the discussion centered around clinical requirements, and the practical (patient and technology) constraints around developing and using a technological device (i.e., wearable device) to monitor and manage spasticity.

It was noted that, the current methods of assessment enable clinicians to judge a degree of limb flexion or velocity, but there are no means to assessment muscle activity, especially in the long-term. Many points were raised, most notably the possibility of using sEMG to assess and monitor spasticity. Research indicates that sEMG can be used to quantify spasticity [37], [38], distinguish between spasticity and dystonia [39], and demonstrate changes in spasticity in patients with post-stroke spasticity [40], [41]. A general consensus was reached that a wearable EMG device capable of longitudinal monitoring, especially when combined with an accelerometer and gyroscope, could provide clinicians and patients with an objective insight into the dynamic course of their condition. The remaining discussion focused on answering specific questions presented below in the results section, focusing on the challenges and requirements in designing and developing a wearable spasticity monitoring device.

## III. Results

### A. How Could a Remote Monitoring Device Help in Managing Spasticity?

If surface EMG is proven to be an adequate surrogate marker of muscle spasticity, and correlates with changes in muscle flexion, there are wide reaching possibilities. A specialist consensus of experts, earlier identified that research should focus on the prevention of contractures, evaluating the outcomes of surgical procedures, and the optimal timing for medical and surgical interventions. Moreover, standardized tools for comparison across specialist centers should be sought [19].

A significant contributing factor as to why there are limited treatment guidelines for spasticity is due to the poor evidence base available for this condition. Researchers would benefit from appropriate, reproducible assessment tools. EMG provides quantifiable data on the degree of a patients’ spasticity, which could allow for the stratification of its severity and be applied towards addressing the efficacy of spasticity treatment modalities. In an ideal setting, this would lead to an improvement in prescribing treatments and generate guidelines that will ultimately improve patient outcomes. Further, the de-prescribing of ineffective medications would reduce unnecessary side effects, polypharmacy, and has cost-saving implications for the health service.

The possibility of remote monitoring also presents additional opportunities. In the U.K., the NHS currently has several promising remote monitoring schemes evaluating conditions such as COPD and frailty [42]. A monitoring device worn by the patient at home sending data to clinicians in hospital about their spasticity could negate the need for frequent hospital visitations. This is particularly beneficial given the strain on resources across NHS trusts. Analysis has shown that virtual appointments can benefit many stakeholders; NHS trusts save millions of pounds, patients save money on travel to and from hospitals, and the environment benefits from reduced CO2 emissions [43]. Methods to remotely assess a patient's condition also enables ongoing data collection and would have the capacity to assess spasticity during activities such as holding a knife and fork when eating or opening a car door and transferring. This could drive treatment to address the problems patients tell us are most problematic, especially given spasticity manifests as a dynamic, progressive condition.

### B. Challenges in the Device Development

Many challenges associated with the development and deployment of a remote spasticity monitoring system was identified alongside the expert panel in Leeds (U.K.). There are technical boundaries to overcome whilst simultaneously making the technology minimally obtrusive and user friendly. The remote monitoring technology system would ideally consist of a wearable device that has integrated sensors, a communication system, and a protocol for the secure transfer of data. This would require a ‘cloud’ infrastructure for secure storage, and software to process and analyze the data generated. The sensor signals, e.g., EMG, from the wearable device must be able to demonstrate, through testing, a high level of reliability and validity. However, even with a high degree of reliability and validity, perhaps the most important consideration in the development of this device is to what extent measurable changes in EMG signals detected by the device correlates with progression or deterioration in a patients’ clinical condition. If the device is sensitive enough to detect encouraging post-intervention EMG changes over time but the patient does not report a corresponding increase in function, then the usefulness of the device is questionable. Careful testing and calibration of the device is required.

The ideal device would be worn and capable of gathering EMG data for 24 hours; this requires a moderately sized, portable power supply. Historically, power supplies for portable biomedical telemetry have been bulky, often requiring the user to wear a backpack connected to the device [44]. Fortunately, as technology has advanced, batteries have decreased in size, last longer, and negate the use of external power supplies. Portable rechargeable batteries have been used to good effect in portable EMG monitors [45] and for sensory control of prosthetic limbs [46] and is likely to be the most sensible approach in the development of this wearable device.

There are many other factors to consider such as biocompatibility and ergonomics. Ideally, patients will manage this device in the community alongside carers when necessary. As such, the device should be as functionally as simple as possible such that minimal clinical or professional support is required. The device should also be sufficiently robust to ensure its longevity. During discussion the question of waterproofing and infection control was raised; the device should be water-resistant during activities of daily living and ideally should be wearable when the patient is participating in self-care activities such as bathing. A practical point raised during the roundtable meeting suggested that if the device was measuring EMG signals over the flexor bulk of the forearm, it should have the capacity to integrate with or be mounted upon a hand resting splint. Finally, health economics around any novel technology requires evaluation. A hybrid design is being considered that enables the re-use of high value components. Cost-effectiveness, while maintaining functionality is crucial. The following paragraphs present specific challenges related to key components of the remote monitoring system.

### C. Challenges in Relation to EMG Data Acquisition and Interpretation

Recent studies have attempted to evaluate the utility of sEMG in quantifying spasticity. However, the use of sEMG signals in quantifying muscle activation, also comes with some known limitations. Specifically, the reliability of sEMG signal reduces with dynamic contractions (limb movements) due to the movement of recording electrodes relative to the skin and underlying muscle. Hence, one of the key challenges will be to establish the type (size, interelectrode distance, material) of surface electrodes that can provide reliable muscle activation patterns for clinically meaningful data that correlate with changes in a patients’ clinical condition. To allow long-term monitoring, the material of the electrode itself must be suitable for wearing for periods over 24 hours without causing adverse skin reactions. Once the sEMG data is acquired, the device needs to have a) preamplifier to filter and amplify signals, b) a filtering unit to filter and further amplify the signal, signal offset and power supply modification, c) analog-to-digital (ADC) conversion and memory unit to store the data. The rechargeable battery powering the device should be of small form factor to be mounted with the wearable device without creating impractical extra weight constraints for the user.

### D. Challenges in Relation to Other Sensory Data Acquisition and Interpretation

Spasticity is a velocity dependent disorder, leading to abnormal changes in the velocity (speed) with which limbs move or do not move. Hence, accelerometry and gyroscope sensors, especially when combined with muscle activation patterns from sEMG can provide a more specific measure of a patients’ activity. Accelerometry and gyroscope data can aid in providing holistic information about the patient's activities, body movement, and inactive periods (sleep). When combined with corresponding muscle activation patterns, it can provide richer and clinically meaningful information about a patients’ spasticity and its relation to the rest of their body movement. Recent research suggests that inertial measurement units can quantify spasticity by analyzing an individual's range of motion [47]. To incorporate accelerometry and gyroscope sensors within the wearable device, the challenge will be to incorporate additional filtering, amplification, ADC and storage units to process and store these signals without drastically increasing energy requirements, and therefore increasing the density of electronic components and cost.

### E. Challenges in Relation to Wireless Data Transfer and Communication Protocols

The wearability of the device imposes constraints on size of each sensing element. There are several options for implementation of monitoring solutions. Almost all of these options present various design trade-offs as detailed below:

#### 1) Smart-watch With Tethered sEMG Sensing

**Fig. 1.**
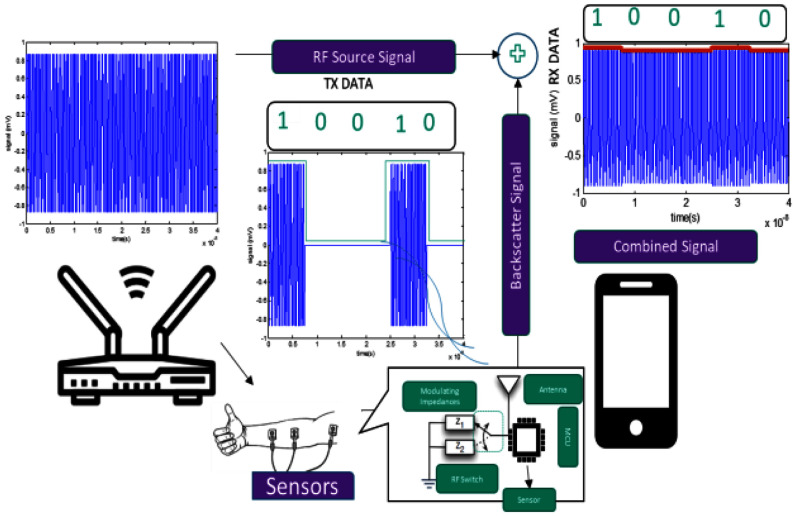
RF Backscatter based system.

A straight-forward option is to have a wearable device, much like a smart-watch which has tethered sEMG sensors. The wearable device can integrate IMUs and other additional sensing modalities e.g., galvanic skin response or Photoplethysmography (PPG) based heart-rate monitoring. The wearable device streams data to an in-home gateway (as shown in Fig. 1, which executes privacy preservation mechanisms before streaming processed data onto cloud systems.

For the in-home connectivity between the wearable device and the home gateway, Bluetooth low energy (BLE) is the first choice for wireless technology. BLE 4.0 provides a theoretical link range of 100 m [48]. Nevertheless, several experimental studies show that actual range can be limited to 10–20 meters in practical deployments [49]. BLE 5.0 provides a coded PHY layer which can theoretically extend the range by five times. However, there are not many indoor studies to verify the actual range of BLE 5.0 in practical deployment. Also increased range is delivered at the cost of increased average current consumption resulting in shorter battery life for wearable devices.

Another possibility is to use sub-GHz connectivity on wearable devices and home gateway. For instance, Long Range Wide Area Network (LoRAWAN) technology provides higher link lengths albeit at lower data-rate and limited duty cycle (1%) for the devices. A thorough comparison of LoRAWAN and BLE 5.0 for indoor monitoring with reliable datasets is still missing from the literature. We plan to address this gap through this project.

#### 2) Wireless Zero-Powered sEMG Sensing

**Fig. 2.**
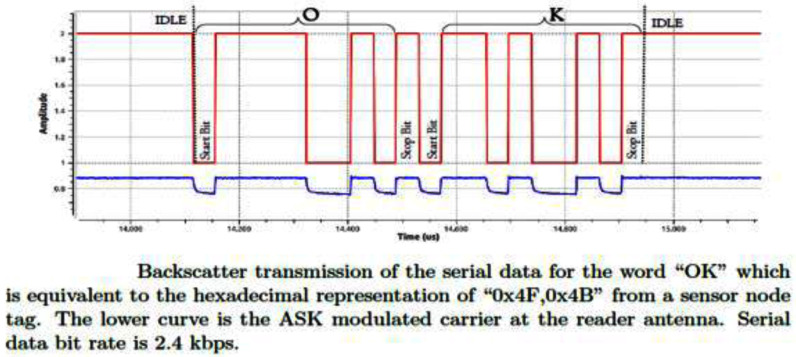
Example of backscatter transmission from [51].

Tethering of the sEMG electrodes limits wearability to a certain extent. Ideally, one wants to design bandage-type, adhesive sensors that are wearable and independently communicate with the gateway. The gateway then performs aggregation and privacy preservation on the collected sensor streams. To that end, one option is to develop Radio Frequency (RF) backscatter [50] based sensors (Fig. 2). Such sensors remodulate ambient RF to encode the sensor data. The two specific challenges in implementation of such sensors are the low data rate and limited range for the sensors. The practical prototype proposed for a different application in our previous project [51] improves upon the range providing 200m link for low data rate applications [52].

However, despite the practical prototypes, there are significant challenges in implementing such devices for in-home monitoring. 1) Multiple data-streams from sEMG sensors need to be multiplexed making load modulation complex. 2) Another design challenge is that in such backscatter systems, gateway is typically a software-defined radio which can implement complex symbol-synchronization and equalization techniques. However, this adds to both cost and complexity during implementation. 3) Unlike BLE based solutions where data can be pushed to the cloud, the RF backscatter-based sensors are more suited to a pull model. In mono-static setup, the gateway must transmit carriers that are then modulated by the device.

In recent times, it has been shown that these sensors can be made more robust by minimizing the footprint of the battery, therefore reducing sensor mass, causing lower levels of motion artifacts [53]. This allows for extended operations, higher data quality and a longer range. It is possible to use certain smartphones as a gateway e.g., as shown in [54]. Frequency Modulation (FM) receivers on mobile phones can also be used for similar purposes [55]. Since commodity FM transceivers are easier to build and are indeed supplied as modules, it is easier to integrate these into a gateway. We plan to explore such designs through this project.

Furthermore security during transmission from any of the methodologies outlined can be encrypted such that attack vectors such as ‘man in the middle’ can be minimized. This is easier to attain when using well-established standards e.g., BLE. With BLE 4. the payload size was limited. However, BLE 5 supports extended and multi-advertisement. Therefore applying SHA-256 on the payload is now feasible. Encryption of data on RF backscattering is more challenging. Mainly due to the limited amount of battery power and compute capability. Many modern MCUs support implementation of techniques such as Elliptic Curve Cryptography (ECC). However, the transmission of ECC payload poses additional energy overhead. In due regards, simple obfuscation of time-series data through symbolic or proprietary coding is more beneficial.

### F. Challenges in Relation to Patient Data Privacy, Safety and Integrity etc.

Patient data privacy is a key issue for implementing the proposed monitoring solution. As indicated in the above discussion, the clinical assessment of the raw data along with patient diaries will enable us to establish baseline data. Once this baseline is established and a sufficient amount of data is collected, the process can be automated to allow edge-processing. For instance, one can implement automated classification using Tiny-Machine Learning (TinyML) [56] on the gateway devices. TinyML is an emerging research area at the intersection of cyber physical systems and machine learning. Effectively, TinyML provides tools to develop ML models which can be executed on resource-limited devices. Employing TinyML, classification using Long-Short-Term-Memory (LSTM) or other Recurrent Neural Networks (RNN) based mechanisms for time series data can be used to generate alarms. Consequently, rather than sharing the raw-data only, alarm information or raw information encoded into class markers can be shared. However, such an approach is only viable when enough data is present which will be at the latter stages of this project. To begin with, informed consent from participants is required and technical architecture should allow withdrawal of such consent as well as transparency to show data is being used for its stated purpose as specified by EU General Data Protection Regulation (GDPR) requirements. A detailed Data Protection Impact Assessment (DPIA) needs to be set up in accordance with guidance from the U.K. Information Commissioner's Office.

### G. Challenges in Relation to Integrating Acquired Data Within the Healthcare/NHS System

Clinical information relating to the care of a patient should be stored in the medical record of that individual so that it is accessible to everyone involved in the individual's care. In the U.K., this record is commonly on the platform of an Electronic Health Record (EHR) system. Patients also have the right to access information held about them, which is facilitated by organizations managing this sensitive personal information on a small number of centralized systems. Longitudinal physiological data monitoring produces a volume of data which is too high to be useful or reasonably stored in its raw form, so a meaningful summary must be produced for sharing and storage in health records. This has a precedent in other common clinical investigations such as ambulatory electrocardiogram monitoring or sleep studies. This requires data transfer between the computer system on which raw data is analyzed to produce a clinically relevant report, and the EHR. In some NHS systems this step is performed manually by clinicians further emphasizing the need to free up clinical time for clinical duties. Integrating systems requires investment upfront in governance to safeguard data security and data sharing between systems, but automating the sharing of data with the EHR may produce more optimal patient care by keeping it reliably up to date and may save time for clinicians.

## IV. Future Directions and Potential Healthcare Impact

As part of this ongoing project: Sensorization to Premeditate and Attenuate Symptoms in the Management of Spasticity (SPASMS), the team (authors), is in the process of developing the proposed wearable monitoring system that aims to longitudinally monitor spasticity in patients’ within the community setting. Following the completion of the above prototype system, first 1) it will be rigorously tested in the laboratory environment in healthy individuals against the current gold standard sEMG and inertial measuring unit (IMU) sensors. Subsequently, following ethical approval, 2) the system will be tested on a small number of stroke patients in clinic and 3) in stroke patients within the home setting. The above chronological order should enable testing of the system in the real world environment along with gaining systematic user feedback to design a truly user friendly monitoring system. It is envisaged that effectiveness and practicalities of this system will be further assessed through clinical trial(s), which will also enable the system to meet required regulatory medical device standards and regulations (UKCA in the U.K.) to be approved as a medical device.

Continuous community-based monitoring of spasticity would bring substantial benefits to patients, their families and carers, clinicians, and health and social care economies. Combining current states-of-the-art across a diverse range of engineering fields can bring us closer to achieving this goal.

Facilitating the pathway to the ‘clinic in the home’, such that patients feel empowered with the tools that can aid them in the management of their healthcare is a key driver in the innovation of healthcare technologies worldwide.

Such a wearable monitoring system has the prospect of managing several conditions outside the remit of managing stroke, spasticity and cerebral palsy. Given multiple pathologies affect muscle activation, there is the potential to track any response to treatment, delineate disease severity or form a cornerstone as part of the rehabilitation process. Real-time feedback could contribute towards a patient's awareness of their muscle function, known to lead to better adherence to therapy and exercises. From a clinical perspective, examples include: a) Multiple Sclerosis (endurance patterns mapping, enabling therapists to design fatigue management programs), b) Parkinson's disease (can guide medication doses, analyze tremor patterns and assess the effectiveness of treatments such as deep brain stimulation), c) Chronic pain such as muscle tension and overuse including Fibromyalgia. We know that EMG biofeedback helps patients to learn how to consciously relax muscles that are overused, and this may provide some benefit. Muscle dystonia causes involuntary muscle contractions and as such botulinum toxin therapy can be evaluated, d) Nerve regeneration after surgical repair or temporary cessation in function from injury can be mapped. Clinicians perform clinical tests (Tinel's), however to provide more objective measure (e.g., shoulder dislocation causing nerve paralysis or brachial plexus injury), wearable monitoring, guided by EMG, can be useful in decision making about whether to manage the injury expectantly or perform invasive surgical procedure, f) Musculoskeletal injuries for example from sports can be helped to guide corrective exercises.

Therefore, although the proposed system is specifically designed and developed for spasticity monitoring, it is envisaged the applications of such a system can be varied where long-term monitoring of muscle activity and movement dynamics is desirable.

## Conflict of Interest

The authors declare that they have no conflict of interest.

## Author Contribution

ANP came up with the initial concept for the technology. KRA and RJO provided initial clinical input. With this clinical input, RH, AS, SRZ and ANP proposed the presented technological solution. ANP acquired the funding. SH led the workshop and provided critical clinical input, aided by KRA, SRS, CW, CH and RJO. Engineering and Technological aspects of this manuscript were led by RH, AS, SRZ and ANP. Clinical aspects of this manuscript were led by RJO, KRA and SH, with input from CW and CH. Evolving manuscript versions were maintained by SRS. Additionally, each author contributed their specialized expertise in their respective research domains to the overall interpretation, discussions and insights offered by the paper, as well as reviewing and editing the manuscript.
